# Mid-infrared Photoconductive Response in AlGaN/GaN Step Quantum Wells

**DOI:** 10.1038/srep14386

**Published:** 2015-09-23

**Authors:** X. Rong, X. Q. Wang, G. Chen, X. T. Zheng, P. Wang, F. J. Xu, Z. X. Qin, N. Tang, Y. H. Chen, L. W. Sang, M. Sumiya, W. K. Ge, B. Shen

**Affiliations:** 1 State Key Laboratory of Artificial Microstructure and Mesoscopic Physics, School of Physics, Peking University, Beijing 100871, China; 2 Collaborative Innovation Center of Quantum Matter, Beijing, China; 3 Laboratory of Semiconductor Material Science, Institute of Semiconductors, CAS, Beijing 100083, China; 4 National Institute for Materials Science (NIMS), Tsukuba, Ibaraki, 305-0044, Japan

## Abstract

AlGaN/GaN quantum structure is an excellent candidate for high speed infrared detectors based on intersubband transitions. However, fabrication of AlGaN/GaN quantum well infrared detectors suffers from polarization-induced internal electric field, which greatly limits the carrier vertical transport. In this article, a step quantum well is proposed to attempt solving this problem, in which a novel spacer barrier layer is used to balance the internal electric field. As a result, a nearly flat band potential profile is obtained in the step barrier layers of the AlGaN/GaN step quantum wells and a bound-to-quasi-continuum (B-to-QC) type intersubband prototype device with detectable photocurrent at atmosphere window (3–5 μm) is achieved in such nitride semiconductors.

III-nitrides are employed in a wide range of applications such as solid-state lighting, display, wireless communication, and so on^1^,^2^,^3^. AlGaN, with bandgap energy tunable from 3.4–6.2 eV, is able to cover the wavelength from deep ultraviolet (DUV, AlN) to ultraviolet (UV, GaN), providing a unique choice for fabricating UV detectors, in particular for solar-blind UV detectors^4^. Moreover, Al(Ga)N/GaN quantum wells (QWs) also provide a capability to realize high performance infrared devices based on intersubband transitions (ISBT)^5^,^6^,^7^,^8^,^9^. Thus, III-nitrides show a unique opportunity to realize single-chip monolithic dual-band UV- and infrared- photodetectors, which will definitely improve the detection ability in a great deal^10^. In addition, AlGaN based materials exhibit large conduction band offset (CBO, ~1.9 eV for AlN/GaN), large LO-phonon energy (~92 meV), short carrier relaxation time (170–370 fs), high-energy remote lateral valleys (>2 eV above Г) and environmental stability as wide-gap semiconductors^11^, and thus show advantages even over the conventional materials for ISBT-based infrared detectors.

For a single-chip monolithic dual-band UV- and infrared- photodetector, it is not hard to fabricate solar-blind UV detectors based on AlGaN p-i-n structure at the present time, the current difficulty lies however in realizing high performance infrared detectors based on III-nitrides, in particular at the 3–5 μm atmosphere window. This difficulty basically originates from two aspects, i.e. the poor crystalline quality and effect of polarization-induced internal electric field^11^,^12^. The former one is the key disadvantage for III-nitrides comparing to conventional semiconductors such as GaAs for infrared detectors and hence requires for long-term effort. This one is probably solved by using high quality GaN free-standing substrate in future. The latter one is also special for III-nitrides, since for which good quality III-nitride materials are generally grown along *c*-axis and inevitably exhibit lattice polarization. The polarization-induced internal electric fields would reduce not only the ISBT oscillator strength but also the vertical photocurrent transport^11^,^13^,^14^. Although this problem could be solved by using epitaxy along non-polar directions, the even poorer crystalline quality of non-polar III-nitrides seriously declines the device performance and is thus still a great challenge in practice^13^.

In this article, we report our efforts on modifying the internal electric fields in the AlGaN/GaN multiple quantum structures for infrared detection at the 3–5 μm atmosphere window. The core idea is to create a nearly flat band potential in the step barrier layers by using step-quantum-wells (step-QWs). Such a flat band potential is much favorable for photocurrent vertical transport through the quasi-continuum (QC) states formed above the step barrier energy, while it still maintains high crystalline quality grown along *c*-axis. Meanwhile the step barrier layers are thick enough (~16 nm) to block dark current through the ground state. Finally an AlGaN-based bound-to-quasi-continuum (B-to-QC) type photodetector with a photocurrent peak at 3.40 μm is achieved at 5 K, in agreement with the photo-absorption measurement.

## Results

**Figure 1:**
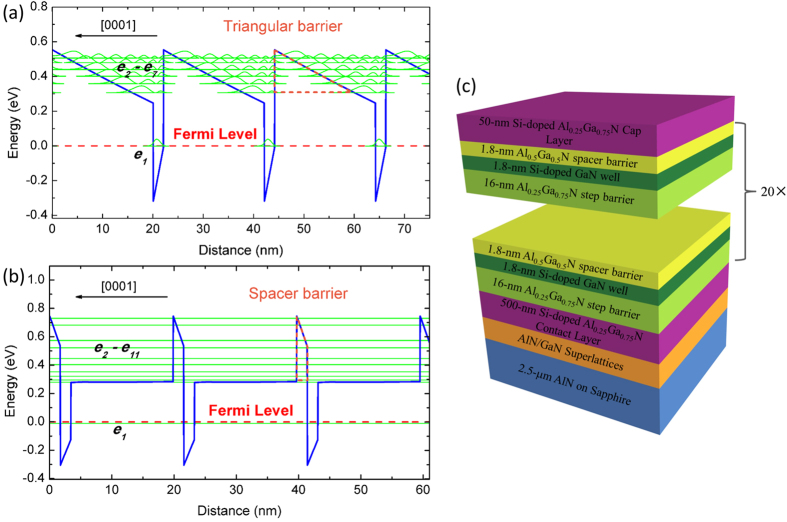
(**a**) Conduction band profile of Al_0.35_Ga_0.65_N/GaN (20 nm/2 nm) multiple-QWs and the triangular barrier (orange dashed line). (**b**) Conduction band profile of Al_0.5_Ga_0.5_N/GaN/Al_0.25_Ga_0.75_N (1.8 nm/1.8 nm/16 nm) step-QWs designed for B-to-QC ISBT and the spacer barrier (orange dashed line). (**c**) Schematic image of the designed sample structure.

Firstly, we designed the sample structures of multiple-QWs and step-QWs by self-consistently solving Schrödinger-Poisson equations (see the Methods section for details). Figure 1(a) shows the schematic conduction band profile and corresponding subbands of a conventional AlGaN/GaN multiple-QWs structure for 3–5 μm infrared detection, which consists of a 20-nm-thick Al_0.35_Ga_0.65_N barrier and a 2-nm-thick Si-doped GaN well in each period. In comparison, Fig. 1(b) shows the new design, i.e. a step-QWs structure, where each period of quantum structure includes a 1.8-nm-thick Al_0.5_Ga_0.5_N spacer barrier, a 1.8-nm-thick Si-doped GaN well and a 16-nm-thick Al_0.25_Ga_0.75_N step barrier. All the barrier layers are unintentionally doped to maintain high resistance for blocking dark current. The key point for the new design is using a novel spacer barrier (orange dashed line) and an Al_0.25_Ga_0.75_N step barrier instead of a conventional Al_0.35_Ga_0.65_N barrier, which is assumed to suppress the polarization effect. As shown in Fig. 1(a), the photocurrent vertical transport in the multiple-QWs structure is seriously blocked by the triangular barriers (orange dashed line) associated with the lattice polarity. In contrast, as shown in Fig. 1(b), a nearly flat band potential in the step barrier layers is created by polarization engineering approach in the step-QWs structure and the excited states from *e*_*2*_ to *e*_*11*_ are coupled to form minibands, defined as quasi-continuum (QC) states. Thus, a B-to-QC type photocurrent transport is expected to realize since the spacer barrier layers are designed narrow enough for the coupling and resonant tunneling. We noticed that a similar structure of step-QWs has been employed previously to realize bound-to-bound (B-to-B) photodetectors at terahertz range^15^,^16^,^17^,^18^,^19^. There is however a major difference that all the subbands including ground state are above the step barrier energy in the previous structure, while in this work the ground state is confined in the quantum wells to realize mid-infrared B-to-QC photodetectors.

The key point in the polarization engineering approach is in fact to modify the Al composition and the strain in the active region. On one hand, the Al composition in the AlGaN step barrier layer is controlled as the same as the average Al composition in the whole step-QWs in order to eliminate the differences of the spontaneous polarization^15^. On the other hand, the strain-engineering is employed to avoid any additional piezoelectric polarization contributions, i.e. the overall strain in the step-QWs is almost fully relaxed^20^,^21^. This is achieved by adopting an AlN/GaN superlattices interlayer between the active region and AlN template and carefully controlling the Al composition in the contact/cap layer as the same as the average Al percentage in the whole step-QWs. These two points are experimentally confirmed (shown later) by high-resolution x-ray diffraction (HR-XRD), reciprocal space mappings (RSMs) and high-resolution transmission electron microscopy (HR-TEM).

The sample was grown by plasma-assisted molecular beam epitaxy (PAMBE, SVTA). The designed sample structure was shown as Fig. 1(c). About a 2.5-μm-thick AlN layer grown by metal-organic vapor phase epitaxy on c-plane sapphire was used as the template. The growth started from an AlN/GaN superlattices interlayer to release the strain between the epitaxial layer and template and to partially eliminate the threading dislocations^22^. A 500-nm-thick n-type Al_0.25_Ga_0.75_N contact layer was then grown, followed by 20 periods of AlGaN/GaN step-QWs. Finally, a 50-nm-thick n-type Al_0.25_Ga_0.75_N cap layer was deposited. The growth was performed under a slightly Ga-rich condition, leading to an atomically flat surface. Reflection high energy electron diffraction (RHEED) was used to *in-situ* monitor the growth process and the RHEED pattern was kept streaky all along the sample growth process. Si was used as the n-type dopant, and the residual electron density in the GaN well was ~1 × 10^19^ cm^−3^.

**Figure 2:**
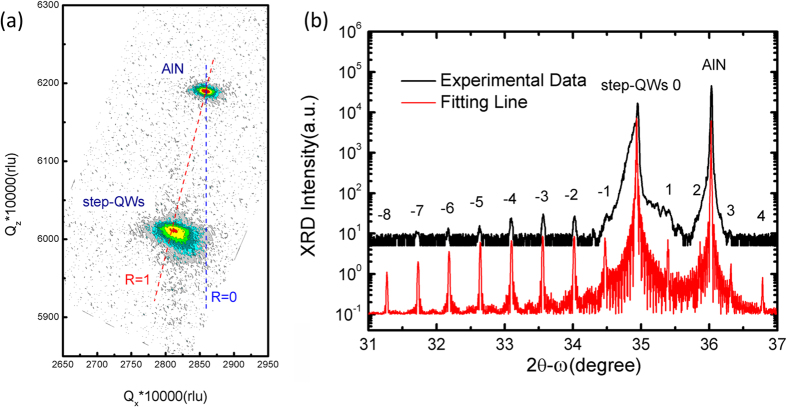
(**a**) Reciprocal space mappings (RSMs) of asymmetric (104) plane for the sample. (**b**) Experimental curve of HR-XRD 2θ-ω scans of symmetric (0002) plane for the sample (black line) and the corresponding simulated curve (red line).

**Figure 3:**
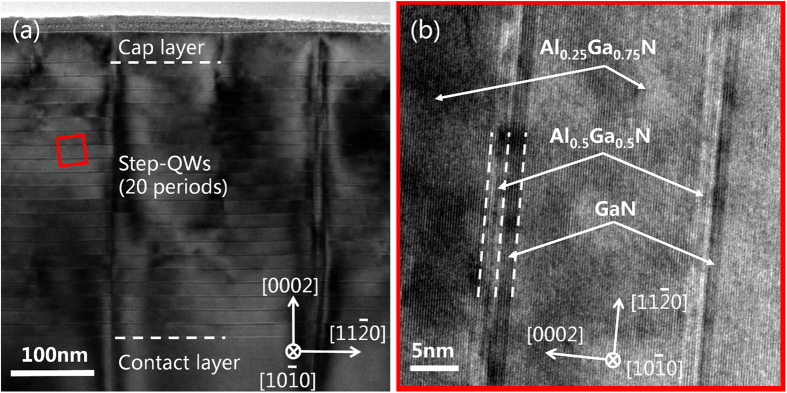
(**a**) Bright field TEM image with g vector of [0002] for the whole step-QWs. (**b**) Cross-sectional HR-TEM image of the area marked with red square in (**a**).

Residual strain of the sample has been investigated by RSMs of asymmetric (104) plane in XRD measurement and the result is shown in Fig. 2(a). As one can see, the relaxation factor *R* of the step-QWs is estimated to be ~95%, which means that the strain in the Al_0.25_Ga_0.75_N step barrier layers is almost fully relaxed since the step barrier layers are the dominant components of the step-QWs. Figure 2(b) shows the HR-XRD 2θ-ω scans of the symmetric (0002) plane and their fitting line for the sample. More than 10 grades of satellite diffraction peaks of the step-QWs are observed, showing that the step-QWs are in excellent periodicity with sharp interfaces. This is further confirmed by the microstructure measurement using HR-TEM. Figure 3(a) shows a cross-sectional bright-field image of the sample with *g* vector of [0002]. Twenty periods of AlGaN/GaN step-QWs as well as the Al_0.25_Ga_0.75_N contact/cap layer can be seen from the image. The zoom-in HR-TEM image for the small region marked with red square in Fig. 3(a) is shown in Fig. 3(b). It is clear that the interfaces of the step-QWs are quite sharp and the ultra-thin wells and spacer layers are clearly observed. The thickness of the Al_0.5_Ga_0.5_N spacer barrier/GaN well/Al_0.25_Ga_0.75_N step barrier estimated by the HR-TEM is about 1.8/1.6/16.7 nm, respectively, in accord with the designed values.

**Figure 4:**
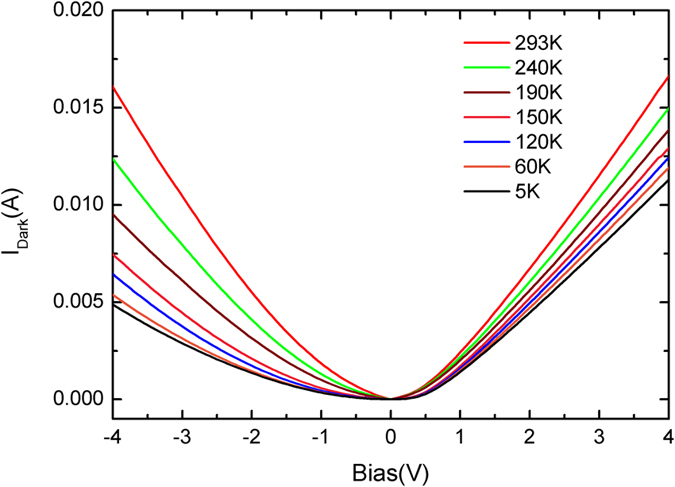
Temperature dependence of the absolute value of dark current on the bias applied for the sample. A symmetric *I-V* characteristic curve at room temperature (RT) and asymmetric ones at low temperatures (<250 K) are observed.

Simulation of the HR-XRD 2θ-ω scans shown in Fig. 2(b) tells that the Al compositions in the spacer barrier and the step barrier are 0.54 ± 0.02 and 0.26 ± 0.02, respectively, in agreement with the designed values. The differences between the experimental results in each layer and the designed ones would result in a variation of ISBT energy, which is estimated to be ~15 meV smaller than the designed one. Further, we notice that the average Al composition in the total step-QWs is about 0.27 ± 0.02, which is close to the value of 0.26 ± 0.02 of the step barrier layers. We can thus well assume that a nearly flat band potential appears in the step barrier layers. This flat band potential can also be illustrated by the temperature dependent dark current measurement, i.e. current-voltage (*I-V*) characteristic curves. As shown in Fig. 4, a symmetric *I-V* characteristic curve at room temperature (RT) and asymmetric ones at low temperatures (<250 K) are observed. There are two sources that should be taken into account for the formation of the dark current, namely the inter-well tunneling and thermionic emission^14^. We have probed which one is the dominant factor of the dark current in our sample (not shown here), and found that the thermionic emission scheme dominates the dark current at RT while the inter-well tunneling is dominant at low temperatures (<250 K). In the former case, the ground state carriers partially jump up to the QC states by thermionic emission at RT. Thus the elimination of the internal electric fields at the step barrier layers will result in a symmetric *I-V* characteristic curve provided that we can ignore the influence of the spacer barrier as it is narrow enough for easy resonant tunneling. In the latter case, however, the ground state carriers are mostly confined in the quantum wells at low temperatures (<250 K). So the carriers have difficulties in tunneling into the adjacent well at low bias because of the wide step barrier blocking. Such an asymmetric structure results in the asymmetric tunneling scheme, and consequently asymmetric *I-V* characteristic curves.

**Figure 5:**
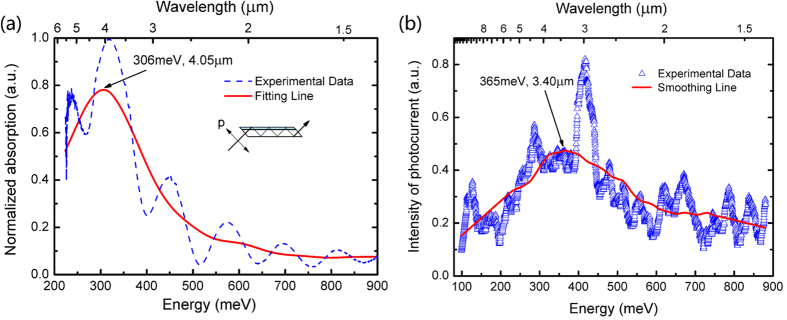
(**a**) Normalized photo-absorption spectrum (blue dashed line) and its corresponding fitting line (red solid line) for the sample (**b**) Photocurrent spectrum measured at 5 K (blue open triangles) and its corresponding smoothing line (red solid line).

To get the photo-absorption and photocurrent response, a wire grid polarizer was used to generate *p*- or *s*-polarized light. The measurement configuration is shown in the inset of Fig. 5(a). According to the optical selection rule, the *p*-polarized light was considered as the signal beam while the *s*-polarized light was regarded as the background^14^. Figure 5(a) shows the experimental photo-absorption spectrum (blue dashed line) and the Gaussian fitting line (red solid line), measured by Fourier transform infrared spectroscopy (FTIR) at RT. The periodic interference peaks in the experimental line are due to the different refractive index between the epitaxial layer and sapphire. A broad photo-absorption peak at 4.05 μm (306 meV) can be seen in the detection range of 1.4–6 μm from the solid line. The absorption spectrum is broad with a full width at half maximum (FWHM) of more than 200 meV, showing that the QC states are indeed created and the absorption just corresponds to ISBT from the ground state *e*_*1*_ to the QC states. Grateful to the nearly flat band potential in the step barrier layers, a photocurrent response is clearly observed at a bias of −1 V at 5 K, as shown in Fig. 5(b). The photocurrent signal with fluctuations (blue open triangles) is smoothed and shown as the smoothing line (red solid line), where a photocurrent peak at ~3.40 μm (365 meV) is identified. In contrast, it is difficult to detect photocurrent signal from conventional multiple-QWs structures.

## Discussion

It is noted that the peak energy of the photocurrent is about 365 meV, which is blue-shifted to about 60 meV compared with that of the photo-absorption peak energy (306 meV). This probably originates from several issues, including the bandgap magnification at 5 K, carrier vertical transport, bias applied, and compressive strain as sapphire exhibits higher thermal expansion than the epitaxial layer. The blue-shift contributed from the bandgap magnification is estimated to be quite small since the bandgap magnification at 5 K (compared with RT for photo-absorption measurement) for the Al_0.26_Ga_0.74_N step barrier is about 5 meV larger than that for the GaN quantum well by the Varshni model^23^,^24^. We will mainly discuss below the contribution of the carrier vertical transport. As is known, the detected photocurrent is determined by two processes in sequence. The first one is the carriers jumping up from the ground state to excited states by absorbing the relevant photons, and the second one is the excited carriers escaping from the quantum wells under a certain vertical bias. The electrons in the GaN quantum wells making transition from the ground state to higher energy QC states would obviously have much higher tunneling probability through the spacer barrier layers, leading to higher photocurrent response, even though the absorption coefficient of ISBT from *e*_*1*_ to *e*_*2*_ (the bottom of the QC states) is the highest. So we come to a conclusion that the photocurrent signal mainly corresponds to ISBT from *e*_*1*_ to higher energy QC states which benefits from both the absorbing process and vertical tunneling. The bias applied and compressive strain will also influence the peak energy of the photocurrent but are estimated not the dominant issues. Considering all the above issues and also the fact that the QC states with an energy width of about 300 meV, the blue-shift being as large as 60 meV is a reasonable result.

It would be interesting to compare the experimental results between the present step-QWs structure investigated in this work and the conventional multiple-QWs structure. The sample with a conventional AlGaN/GaN multiple-QWs structure has been studied in our previous work^14^. The relatively large dark current in the present sample compared with the conventional one probably comes from the device fabrication process. The fact of the obvious photoconductive response obtained based on the present structure proves with no doubt that the nearly flat band potential in the novel step-QWs structure is very favorable for obtaining photocurrent signal. Another exciting result is that not multi-bands photoconductive response as shown in ref. 14 but rather a single photocurrent peak is observed in the present structure as a result of the B-to-QC ISBT. In addition, a further design can be adopted to upgrade the step-QWs structure considering that the present structure lowers down the wave function overlap between the ground state and QC states and causes an even lower ISBT probability. This shortcoming should not be neglected even though the nearly flat band potential brings tremendous benefits by realizing a B-to-QC type photocurrent transport. And the further design can balance these issues by using two Al_0.5_Ga_0.5_N spacer barrier layers, i.e. using the structure of Al_0.5_Ga_0.5_N/n-type GaN/Al_0.5_Ga_0.5_N/Al_0.25_Ga_0.75_N with each layer thickness of about 1/2/1/20 nm. Thus, a nearly flat band potential in the Al_0.25_Ga_0.75_N step barrier layers can be created, and meanwhile the ISBT probability can be maintained high.

In conclusion, we designed a new type of AlGaN/GaN step-QWs structure for ISBT infrared detection towards 3–5 μm and high quality sample with ultra-sharp interfaces has been grown on c-plane sapphire. A nearly flat band potential in the step barrier layers is created, which is an efficient approach to eliminate the adverse influence of the polarization effect. This new structure is proved to be very favorable for the creation of B-to-QC type photocurrent vertical transport. As a result, photoconductive response is successfully detected peaking at 3.40 μm when measured at 5 K, at that temperature the dark current of which the inter-well tunneling being the dominant scheme is well blocked. The photoconductive response peak is about 60 meV blue-shifted in comparison with that of the photo-absorption, and we suggest that the main reason of this shift is that the photocurrent signal corresponds to ISBT from *e*_*1*_ to higher energy QC states. Our work demonstrates that the step-QWs structures have great potential in applications for mid-infrared detection.

## Methods

### Numerical simulations

The self-consistent Schrödinger-Poisson equations assumed periodic potential and charge neutrality conditions. In the calculation, the bandgap energy was chosen to be 3.4 eV (6.1 eV) for GaN (AlN), and the bowing parameter for AlGaN was set as 1 eV. The ratio of CBO to band offset was set as 70%. The lattice constants, spontaneous polarization and piezoelectric constants of AlN and GaN were taken from ref. 12, and we assumed the structure strained on the Al_0.25_Ga_0.75_N layers. The thickness of GaN quantum wells was carefully determined so that only the ground state, marked as *e*_*1*_, was confined in the GaN wells. The ISBT energy between *e*_*1*_ and *e*_*2*_ was designed as ~300 meV, necessary to realize an infrared detection towards 3–5 μm atmosphere window.

### Measurements

The crystalline quality of the sample was characterized by HR-XRD (Bruker D8) and HR-TEM (Tecnai F30). Carrier concentration was investigated by a Hall-effect measurement system (ACCENT-HL5500). For the electrical measurements, 200 μm × 200 μm mesa structures were first fabricated and then a metallization scheme of Ti/Al/Ni/Au with thickness of 20/175/40/500 nm was deposited on the Al_0.25_Ga_0.75_N contact/cap layers. For the infrared measurements, the sample was mechanically polished into 45° waveguides. The photo-absorption spectrum was measured by FTIR (Bruker IFS120). The photocurrent spectrum and the temperature dependent dark current were investigated by another FTIR system (Nicolet 6700).

## Additional Information

**How to cite this article**: Rong, X. *et al.* Mid-infrared Photoconductive Response in AlGaN/GaN Step Quantum Wells. *Sci. Rep.* **5**, 14386; doi: 10.1038/srep14386 (2015).

## References

[b1] Ponce F. A. & Bour D. P. Nitride-based semiconductors for blue and green light-emitting devices. Nature 386, 351 (1997).

[b2] DenBaars Steven P. *et al.* Development of gallium-nitride-based light-emitting diodes (LEDs) and laser diodes for energy-efficient lighting and displays. Acta Mater 61, 945 (2013).

[b3] Tansu N. *et al.* III-nitride photonics. IEEE Photonics J 2, 241 (2010).

[b4] Tut T. *et al.* Solar-blind AlGaN-based p-i-n photodetectors with high breakdown voltage and detectivity. Appl. Phys. Lett. 92, 103502 (2008).

[b5] Suzuki N. & lizuka N. Effect of polarization field on intersubband transition in AlGaN/GaN quantum wells. Jpn. J. Appl. Phys. 38, L363 (1999).

[b6] Vardi A. *et al.* Near infrared quantum cascade detector in GaN/AlGaN/AlN heterostructures. Appl. Phys. Lett. 92, 011112 (2008).

[b7] Gmachl C. *et al.* Intersubband absorption at λ~1.55 μm in well- and modulation-doped GaN/AlGaN multiple quantum wells with superlattice barriers. Appl. Phys. Lett. 77, 3722 (2000).

[b8] Machhadani H. *et al.* GaN/AlGaN intersubband optoelectronic devices. New J. Phys. 11, 125023 (2009).

[b9] Baumann E. *et al.* Near infrared absorption and room temperature photovoltaic response in AlN/GaN superlattices grown by metal-organic vapor-phase epitaxy. Appl. Phys. Lett. 89, 041106 (2006).

[b10] Hofstetter D. *et al.* Monolithically integrated AlGaN/GaN/AlN-based solar blind ultraviolet and near-infrared detectors. Electron. Lett. 44, 986 (2008).

[b11] Feezell D. *et al.* Optical properties of nonpolar III-nitrides for intersubband photodetectors. J. Appl. Phys. 113, 133103 (2013).

[b12] Bernardini F. *et al.* Spontaneous polarization and piezoelectric constants of III-V nitrides. Phys. Rev. B 56, R10024 (1997).

[b13] Pesach A. *et al.* Non-polar m-plane intersubband based InGaN/(Al)GaN quantum well infrared photodetectors. Appl. Phys. Lett. 103, 022110 (2013).

[b14] Chen G. *et al.* Multi-bands photoconductive response in AlGaN/GaN multiple quantum wells. Appl. Phys. Lett. 104, 172108 (2014).

[b15] Beeler M. *et al.* Terahertz absorbing AlGaN/GaN multi-quantum wells: Demonstration of a robust 4-layer design. Appl. Phys. Lett. 103, 091108 (2013).

[b16] Machhadani H. *et al.* Terahertz intersubband absorption in GaN/AlGaN step quantum wells. Appl. Phys. Lett. 97, 191101 (2010).

[b17] Wu F. *et al.* Terahertz intersubband transition in GaN/AlGaN step quantum well. J. Appl. phys. 113, 154505 (2013).

[b18] Sudradjat Faisal F. *et al.* Far-infrared intersubband photodetectors based on double-step III-nitride quantum wells. Appl. Phys. Lett. 100, 241113 (2012).

[b19] Tang C. *et al.* Influence of delta doping on intersubband transition and absorption in AlGaN/GaN step quantum wells for terahertz applications. Physica E 69, 96 (2015).

[b20] Kandaswamy P. K. *et al.* GaN/AlN short-period superlattices for intersubband optoelectronics: A systematic study of their epitaxial growth, design, and performance. J. Appl. Phys. 104, 093501 (2008).

[b21] Beeler M. *et al.* III-nitride semiconductors for intersubband optoelectronics: a review. Semicond. Sci. Technol. 28, 074022 (2013).

[b22] Zhang J. P. *et al.* Crack-free thick AlGaN grown on sapphire using AlN/AlGaN superlattices for strain management. Appl. Phys. Lett. 80, 3542 (2002).

[b23] Tiginyanu I. M. *et al.* Luminescence of GaN nanocolumns obtained by photon-assisted anodic etching. Appl. Phys. Lett. 83, 1551 (2003).

[b24] Silveira E. *et al.* AlN bandgap temperature dependence from its optical properties. J. Cryst. Growth 310, 4007 (2008).

